# Subversion of Host Innate Immunity by *Rickettsia australis via* a Modified Autophagic Response in Macrophages

**DOI:** 10.3389/fimmu.2021.638469

**Published:** 2021-04-12

**Authors:** Jeremy Bechelli, Claire S. Rumfield, David H. Walker, Steven Widen, Kamil Khanipov, Rong Fang

**Affiliations:** ^1^ Department of Pathology, University of Texas Medical Branch at Galveston, Galveston, TX, United States; ^2^ Department of Biological Sciences, Sam Houston State University, Huntsville, TX, United States; ^3^ Laboratory of Tumor Immunology and Biology, National Cancer Institute, National Institutes of Health, Bethesda, MD, United States; ^4^ Center for Biodefense and Emerging Infectious Diseases, University of Texas Medical Branch, Galveston, TX, United States; ^5^ Department of Biochemistry & Molecular Biology, University of Texas Medical Branch at Galveston, Galveston, TX, United States; ^6^ Department of Pharmacology and Toxicology, University of Texas Medical Branch at Galveston, Galveston, TX, United States

**Keywords:** *Rickettsia*, mTOR signaling, autophagy, macrophages, innate immunity

## Abstract

We recently reported that the *in vitro* and *in vivo* survivals of *Rickettsia australis* are *Atg5*-dependent, in association with an inhibited level of anti-rickettsial cytokine, IL-1β. In the present study, we sought to investigate how *R. australis* interacts with host innate immunity *via* an *Atg5*-dependent autophagic response. We found that the serum levels of IFN-γ and G-CSF in *R. australis*-infected *Atg5^flox/flox^*Lyz-*Cre* mice were significantly less compared to *Atg5^flox/flox^* mice, accompanied by significantly lower rickettsial loads in tissues with inflammatory cellular infiltrations including neutrophils. *R. australis* infection differentially regulated a significant number of genes in bone marrow-derived macrophages (BMMs) in an *Atg5*-depdent fashion as determined by RNA sequencing and Ingenuity Pathway Analysis, including genes in the molecular networks of IL-1 family cytokines and PI3K-Akt-mTOR. The secretion levels of inflammatory cytokines, such as IL-1α, IL-18, TNF-α, and IL-6, by *R. australis-*infected *Atg5^flox/flox^*Lyz-*Cre* BMMs were significantly greater compared to infected *Atg5^flox/flox^* BMMs. Interestingly*, R. australis* significantly increased the levels of phosphorylated mTOR and P70S6K at a time when the autophagic response is induced. Rapamycin treatment nearly abolished the phosphorylated mTOR and P70S6K but did not promote significant autophagic flux during *R. australis* infection. These results highlight that *R. australis* modulates an *Atg5*-dependent autophagic response, which is not sensitive to regulation by mTORC1 signaling in macrophages. Overall, we demonstrate that *R. australis* counteracts host innate immunity including IL-1β-dependent inflammatory response to support the bacterial survival *via* an mTORC1-resistant autophagic response in macrophages.

## Introduction

Rickettsiae are Gram-negative, obligately intracellular bacteria transmitted to humans by arthropod vectors. Highly virulent rickettsial species, such as *Rickettsia rickettsii*, can cause life-threatening disease with fatality as high as 40% (1). Clinically, rickettsial illnesses often present with fever, headache, and petechial rash. Although microvascular endothelial cells are the primary target of rickettsial infection (2), rickettsiae effectively invade macrophages and other types of cells, such as dendritic cells and hepatocytes. We, and others, have recently demonstrated that *Rickettsia* invades and survives in human macrophages, while macrophages play an important role in the pathogenesis of rickettsioses (3–9). However, it remains poorly understood how virulent rickettsiae utilize macrophages to develop a systemic infection in mammalian hosts.

Autophagy is an intracellular, bulk degradation process in which a portion of a cytoplasmic component of the cell is engulfed in double-membrane structures known as autophagosomes and is subsequently degraded upon fusion with lysosomes (10, 11). *Atg5* (autophagy-related gene 5) is essential for autophagic vesicle formation as part of the ATG12-ATG5-ATG16 complex (12). A number of studies have employed *Atg5*-conditional knockout mice to investigate the interactions of autophagy with pathogenic microbes (13–15). *Atg5^flox/flox^* Lyz-*Cre* mice were generated by breeding *Atg5 ^flox/flox^* mice to mice expressing the *Cre* recombinase from the endogenous lysozyme M locus (14), leading to an autophagy deficit mainly in macrophages resulting from deletion of the ATG5 gene. The mechanistic (formerly “mammalian”) target of rapamycin (mTOR) is an atypical serine-threonine protein kinase that plays a critical role in maintaining a balance between cellular anabolism and catabolism (16). The mTOR affects several aspects of cellular functions, including metabolism, aging, growth, apoptosis, and autophagy (17). It forms two complexes, mTOR complex 1 (mTORC1) and mTOR complex 2 (mTORC2), with distinct composition and function (18). Toll-like receptor activation, cytokines, and low concentrations of amino acids will activate mTORC1 signaling leading to the phosphorylation of p70S6 kinase (p70S6K), mTOR and S6 kinase, which are established markers of mTORC1 activation (19–22). The mTORC1 is a master regulator of autophagy, since inhibition of mTORC1 was required to initiate the autophagy process (16, 23). Increasing evidence shows that mTORC1 has been implicated in the regulation of fusion of autophagosome to lysosome and the termination of autophagic flux (16).

Although autophagy has been considered a cornerstone of intracellular surveillance and host defense, intracellular bacteria and viruses are known to subvert or modify autophagy to facilitate their infection course (24–26). Recently, we have demonstrated that *R. australis* accumulates and co-localizes with LC3 (+) autophagosomes in macrophages, but not accompanied by a significantly reduced level of SQSTM1/p62 (4). In addition, pharmacological inhibition of mTOR signaling promotes the survival of *R. australis* in macrophages (4). Thus, *R. australis* induces a modified autophagic response, instead of autophagic flux. Using *Atg*5 *^flox/flox^* Lyz-*Cre* and *Atg*5 *^flox/flox^* mice, our recent studies clearly demonstrated that: 1) *R. australis* supports its infection in macrophages by inhibiting anti-rickettsial effect mediated by IL-1β *via Atg5*-dependent autophagic response; 2) *Atg5*-dependent autophagic response in macrophages facilitates the systemic infection of *R. australis* in association with suppressed serum levels of IL-1β (4). Notably, it is unknown how *R. australis* subverts the elements of the host innate immune system to support rickettsial infection *via* a modified autophagic response. Thus, we determined whether inflammatory and anti-inflammatory cytokines other than IL-1β were regulated by *Atg5*-dependent autophagic response *via* utilizing *Atg*5 *^flox/flox^* and *Atg*5 *^flox/flox^* Lyz-*Cre* mice. In addition, we investigated the interactions of *R. australis* with mTOR and its effect on the autophagic response in macrophages. Our studies demonstrate that *R. australis* induces a modified autophagic response while activating mTORC1 signaling in macrophages. Our findings suggest that *R. australis* subverts host innate immunity to support rickettsial infection in association with *Atg5*-dependent regulation of G-CSF and IFN-γ, in addition to IL-1β.

## Materials and Methods

### *Rickettsiae* and Mice

*Rickettsia australis* (Cutlack strain) was cultivated in Vero cells and purified by either Renografin density gradient centrifugation or using a Renografin “cushion” as previously described (27–29) for use in *in vitro* infections. The concentration of rickettsiae propagated in cell culture was determined by plaque assay after purification as described previously (28). Rickettsial stocks were stored at -80°C until use. All the experiments described in this study were conducted in a certified biosafety level 3 (BSL3) laboratory at UTMB. Wild type (WT) B6 mice were purchased from The Jackson Laboratory (catalog number 000664). *Atg*5 *^flox/flox^* Lyz-*Cre* (autophagy deficient), and *Atg*5 *^flox/flox^* (control) mice were kindly provided by Dr. Noboru Mizushima at the University of Tokyo and Dr. Herbert Virgin IV at Washington University School of Medicine in St. Louis (14, 15, 30). For *in vivo* experiments, mice were maintained and handled in a certified animal biosafety level-3 (ABSL3) facility at UTMB and inoculated intravenously (i.v.) through the tail vein with *R. australis* at the doses indicated. *R. australis* used in animal studies was grown in embryonated chicken egg yolk sac culture as described previously (31). Animals were monitored daily for signs of illness and sacrificed at indicated times. *In vivo* experiments were performed according to the Guide for the Care and Use of Laboratory Animals guidelines and approved by the Institutional Animal Care and Use Committee at UTMB.

### Generation of Bone Marrow-Derived Macrophages

Primary bone marrow-derived macrophages (BMMs) were generated from 6-8 week old female WT B6 mice, *Atg*5 *^flox/flox^* mice and *Atg*5 *^flox/flox^* Lyz-*Cre* mice as previously described (4, 14, 15, 30, 32). Briefly, femurs and tibias were dissected, bone marrow was flushed using sterile medium, and cells were cultivated in low-endotoxin DMEM containing 10% (v/v) fetal bovine serum (FBS; Hyclone, Utah, SV30160) supplemented with either 20% supernatant from L929 cell culture or recombinant M-CSF (PeproTech, NJ, 315-02) at 37 °C in 5% CO_2_. Cells were harvested on day 6 and characterized by flow cytometric analysis by staining with anti-F4/80 and CD11b antibodies. Cultures were used when approximately 90% of these cells stained positive for F4/80 and CD11b. BMMs were plated at a density of 1 × 10^6^ cells/well in 24-well plates in RPMI 1640 containing 10% FBS, and experiments were initiated within 24 hrs.

### Macrophage *In Vitro* Infections

Primary mouse BMMs were infected with *R. australis* at a multiplicity of infection (MOI) of 5. In order to synchronize the internalization of bacteria, rickettsiae were centrifuged onto the cells at 560 × g for 5 min and incubated at 37°C in 5% CO_2_. Cells were collected and washed for further experiments at indicated times for each experiment, and uninfected cells served as the negative control.

### Immunoblotting of Molecules Involved in Autophagy and mTOR Signaling

To assess the conversion of LC3-I to the lipidated LC3-II form, cells were lysed with RIPA lysis buffer (EMD Millipore, MA, 20-188) containing protease and phosphatase inhibitors (Roche, IN). Cell lysates were centrifuged to obtain soluble proteins, separated by SDS-PAGE, transferred to polyvinylidene difluoride (PVDF) membranes and probed with polyclonal antibodies against LC3B (Cell Signaling Technology, MA, 4108). Bands were visualized using appropriate secondary antibodies and enhanced chemiluminescence (ECL) detection reagents (Thermo Scientific, Pierce, IL, 32106). Beta-actin was used to determine equal loading of the gel and was detected with mouse monoclonal antibody (mAb, Sigma, MO, A1978). Blotting against SQSTMI was performed using antibodies directed against SQSTM1 (Cell Signaling Technology, MA, 5114). Antibodies directed against phospho-mTOR (Ser2448) (D9C2) XP^®^ rabbit mAb and phospho-p70 S6 Kinase (Thr389) (1A5) mouse mAb (Cell Signaling Technology, MA) were used for the analysis of the mTOR signaling pathway according to the manufacturer’s instructions. Densitometric analysis was quantitatively measured using Image J (33).

### Immunofluorescence Microscopy

For immunofluorescence detection of LC3 puncta in *R. australis*-infected BMMs, cells were first seeded on glass coverslips in 12-well plates one day before infection as described previously (4). At 1 h post-infection (p.i.), cells were washed with PBS, fixed with 4% paraformaldehyde in PBS for 20 min, permeabilized with 0.5% Triton-X in PBS for 20 min and blocked with 3% BSA in PBS for 30 min. Samples were incubated with rabbit monoclonal antibody against LC3B (Cell Signaling Technology, #3868) followed by appropriate secondary antibody. Nuclei were stained with DAPI in ProLong^®^ Gold Antifade Mountant (Life Technology, NY, P-36931). Coverslips were sealed with nail polish, and visualized by confocal microscopy with a 20 × lens (Olympus Fluoview 1000) using FV10-ASW software (Olympus, PA).

The levels of LC3 in cells were quantified as previously described (34). In brief, using Image J, an outline was drawn around each cell and circularity, area, mean fluorescence measured along with several adjacent background readings. The total corrected cellular fluorescence (TCCF) = integrated density – (area of selected cell × mean fluorescence of background readings) was calculated. This TCCF was then equalized against the mean TCCF of neighboring interphase cells in the same field of view, with results presented as fold increase over interphase levels. Box plots and statistical analysis (2-sided unpaired Student t tests) were performed using GraphPad Prism 5.

### Pharmacological Inhibition of mTORC1 Signaling

Rapamycin, the pharmacologic gold standard for inhibiting mTOR, which acts by associating with FK-506 binding protein 12, was used to selectively inhibit mTORC1 (35). To inhibit mTORC1 signaling, cells were treated with 50 ng/mL of rapamycin (Sigma-Aldrich, St. Louis MO) for 4 hours prior to infection with rickettsiae. The cell number was counted, morphology observed by microscopy, and cell viability determined with the trypan blue dye exclusion method (36). The inhibitory effect of rapamycin on mTOR was examined by immunoblotting with antibodies against phospho-mTOR (Ser2448) (D9C2) XP^®^ rabbit mAb and phospho-p70 S6 Kinase (Thr389) (1A5) mouse mAb (Cell Signaling Technology, MA).

### Measurement of Cytokines by ELISA

Supernatants from *Rickettsia*-infected cells and uninfected controls were filter-sterilized and stored as aliquots at - 80°C. The concentration of IL-18 present in the supernatant of *R. australis*-infected BMMs was measured by ELISA using the Mouse IL-18 ELISA Kit (MBL International Corporation), following the manufacturer’s instructions. The detection limit for the ELISA cytokine concentrations was 25 pg/mL for IL-18. Samples were assayed in duplicate and are presented as the average of two independent experiments. Absorbance values were obtained using a VersaMax ELISA microplate reader (Molecular Devices, Sunnyvale, CA), and the concentrations were calculated from values obtained within the linear range of the standard curve.

### Bio-plex Assay for Cytokine Analysis

Cell culture supernatants and mouse sera were processed according to the manufacturer’s instructions and then analyzed using a Bio-plex 200 system (Bio-Rad, Hercules, CA). Briefly, the samples were filter sterilized and subsequently centrifuged for 10 minutes at 450 × g at 4°C to remove debris. The resulting supernatants were collected and aliquoted into 96-well plates and processed for analysis on the Bio-plex system. The cytokines were coupled to cytokine-specific multi-plex beads (Bio-Rad) in the Bio-Plex mouse cytokine immunoassay following the manufacturer’s instructions. The pre-designed assay kit measured the concentrations of cytokines/chemokines including interleukin (IL)-1α, IL-6, IL-10, IFN-γ, granulocyte colony-stimulating factor (G-CSF), and tumor necrosis factor (TNF)-α.

### Next-Generation Sequencing (NGS) and Pathway Analysis

RNA-seq analysis (next-generation sequencing) was performed on *R. australis*-infected BMMs of *Atg5 ^flox/flox^* mice and *Atg5 ^flox/flox^* Lyz-*Cre* mice at 24 h p.i. as described previously (37). Briefly, 1 μg of total RNA from uninfected and infected BMMs was poly A+ selected and fragmented using divalent cations and heat (94°C, 8 min). Illumina TruSeq v2 sample preparation kits (Illumina Inc., San Diego, CA) were used for the RNA-Seq library construction. NGS was performed at the NGS core facility, Sealy Center for Molecular Medicine, the University of Texas Medical Branch (UTMB). Sample libraries were sequenced by the Illumina HiSeq 1500 using a 2 × 50 base paired-end run protocol, with TruSeq v3 sequencing-by-synthesis chemistry. Reads were aligned to the mouse GRCm38 reference genome using the STAR alignment program, version 2.5.3a, with the recommended ENCODE options. GFOLD V1.1.4 was used to calculate the fold change differences between treatments. GFOLD generalizes the fold change by considering the posterior distribution of log fold change to overcome the problem of no replicate samples. Heatmap and hierarchical cluster analysis were generated to demonstrate the expression patterns of the top 100 genes differentially expressed. The dataset was filtered for a Log2 (fold change) ≥ 2.5 or ≤ -2.5 and then uploaded to Ingenuity Pathway Analysis (IPA) software (Ingenuity Systems, Redwood City, CA) for further analysis of the *Atg5*-dependent regulation of transcriptome profile of mouse macrophages during *R. australis* infection.

### Histopathological Analyses

Formalin-fixed, hematoxylin and eosin (H&E)-stained tissue sections from infected and uninfected *Atg*5 *^flox/flox^* and *Atg*5 *^flox/flox^* Lyz-*Cre* mice were evaluated by a pathologist *via* both low-magnification and high-magnification microscopy. Images were taken using an Olympus BX41 photomicroscope (Olympus America, Inc., Center Valley, PA) or using a Revolution Microscope and an iPad Pro^®^ tablet (Echo Laboratory, San Diego, CA). The histopathology slides were read by a board-certified Pathologist who has extensive experience with pathological analysis of rickettsial infections.

### Statistical Analysis

The one-way analysis of variance (ANOVA) with Bonferroni’s procedure was used for comparisons of multiple experimental groups, and a Student t-test or Welch’s t-test was used for two-group comparisons depending on whether the variance between two groups was significantly different. The statistical analyses were performed using GraphPad Prism software version 5.01. *p* values of 0.05 or less were the threshold for statistical significance.

## Results

### *R. australis* Subverts Host Innate Immunity in Association With an *Atg5*-Dependent Autophagic Response

**Figure 1:**
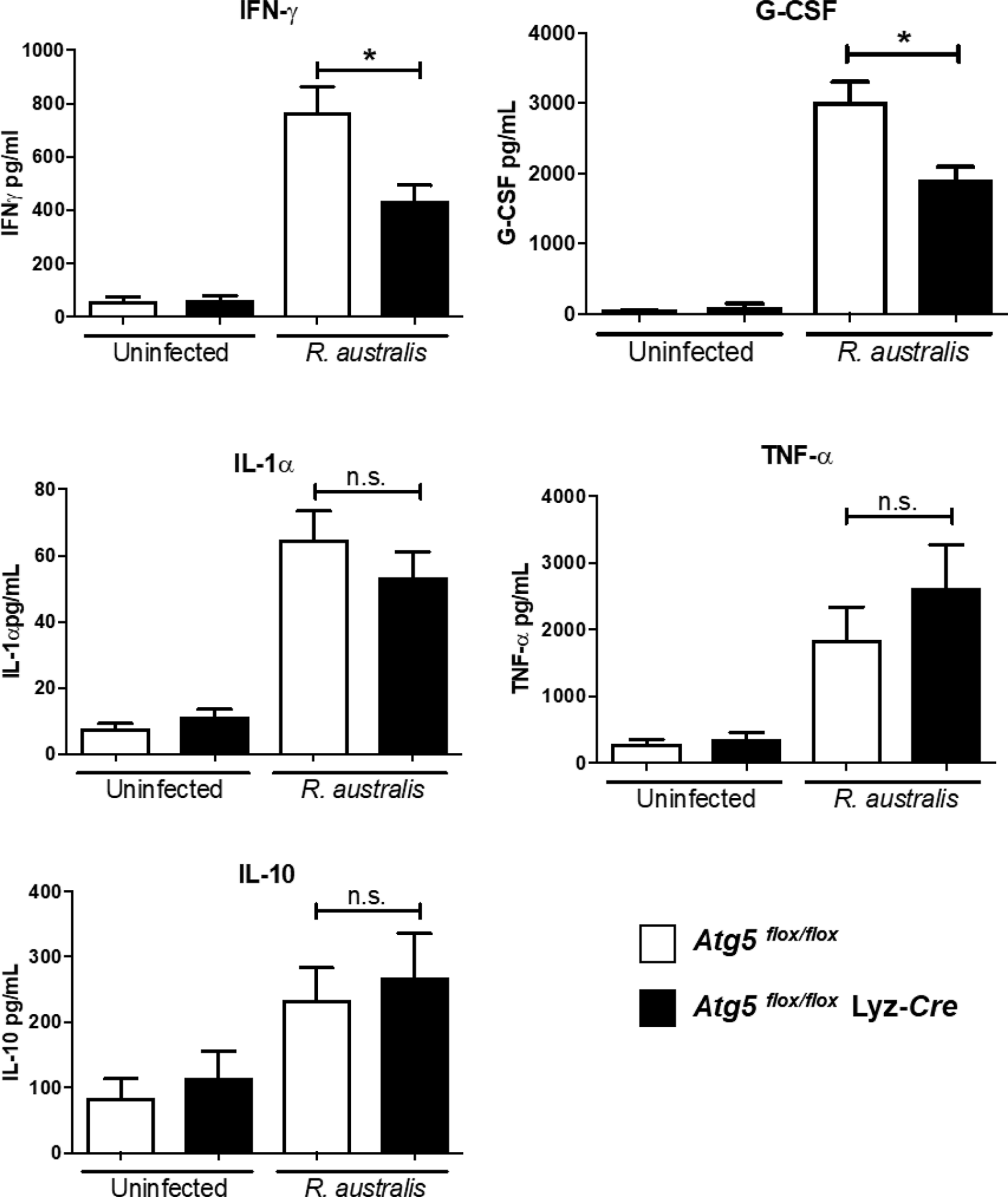
(previous Figure 6). *R. australis* subverts host innate immunity against rickettsioses *via Atg5*-dependent autophagic response. *Atg5 ^flox/flox^* Lyz-*Cre* and *Atg5 ^flox/flox^* mice were infected with *R. australis* i.v. at a dose of 3 × 10^5^ PFU per mouse. On day 4 p.i., mice were euthanized, and serum was collected. Systemic production levels of cytokines/chemokines including IFN-γ, G-CSF, TNF-α, IL-1α, and IL-10 in mouse serum were determined by Bioplex assay. Results are means ± SE of data from three independent experiments containing 4-6 mice per group. *p<0.05; n.s., not statistically significant.

We have previously reported that a type 1 immune cytokine profile is closely associated with the host protective immunity against rickettsial diseases (38, 39). MyD88-dependent cytokines, including IFN-γ, IL-6, IL-12, and IL-1β, are the early signatures of a protective host innate immune response against *R. australis* (38). Inhibiting systemic production of IL-1β by *Atg5*-dependent autophagic response in macrophages contributes to the enhanced *R. australis* infection *in vivo*, as evidenced by our previous published studies employing *Atg*5 *^flox/flox^* Lyz-*Cre* and *Atg*5 *^flox/flox^* mice (4). The important question in this study is whether other components in host innate immunity, in addition to IL-1β, were subverted by *R. australis* to support rickettsial infection *in vivo* in an *Atg5*-dependent mechanism. To answer this question, we determined the association of the *Atg5*-dependent autophagic response with four pro-inflammatory cytokines, IL-1α, TNF-α,IFN-γ and G-CSF, and one anti-inflammatory cytokine, IL-10, in host innate immunity against *R. australis*. First, no significant difference in cytokine levels was identified in uninfected *Atg5 ^flox/flox^* Lyz-*Cre* and *Atg5 ^flox/flox^* mice (**Figure 1**). On day 4 p.i., a time point in host innate immunity at which serum levels of IL-1β and *R. australis* load in tissues were determined in our previous studies, the systemic production levels of IL-1α, TNF-α, and IL-10 in *R. australis*-infected *Atg5 ^flox/flox^* Lyz-*Cre* mice were not significantly different from those in infected *Atg5 ^flox/flox^* mice (**Figure 1**). Surprisingly, serum levels of IFN-γ and G-CSF in *Atg5 ^flox/flox^* Lyz-*Cre* animals were significantly reduced in association with a previously documented lower *R. australis* load in tissues (4), compared to *Atg5 ^flox/flox^* animals (**Figure 1**). Therefore, our results suggest that *R. australis* modulates *Atg5*-dependent regulation of G-CSF and IFN-γ, in addition to IL-1β, in host innate immunity to support rickettsial infection *in vivo*.

### Inflammatory Cellular Accumulation Upon Infection With *R. australis* in Tissue of *Atg5 ^flox/flox^* Mice Is Quantitatively and Qualitatively Different Compared to *Atg5 ^flox/flox^* Lyz-*Cre* Mice

**Figure 2:**
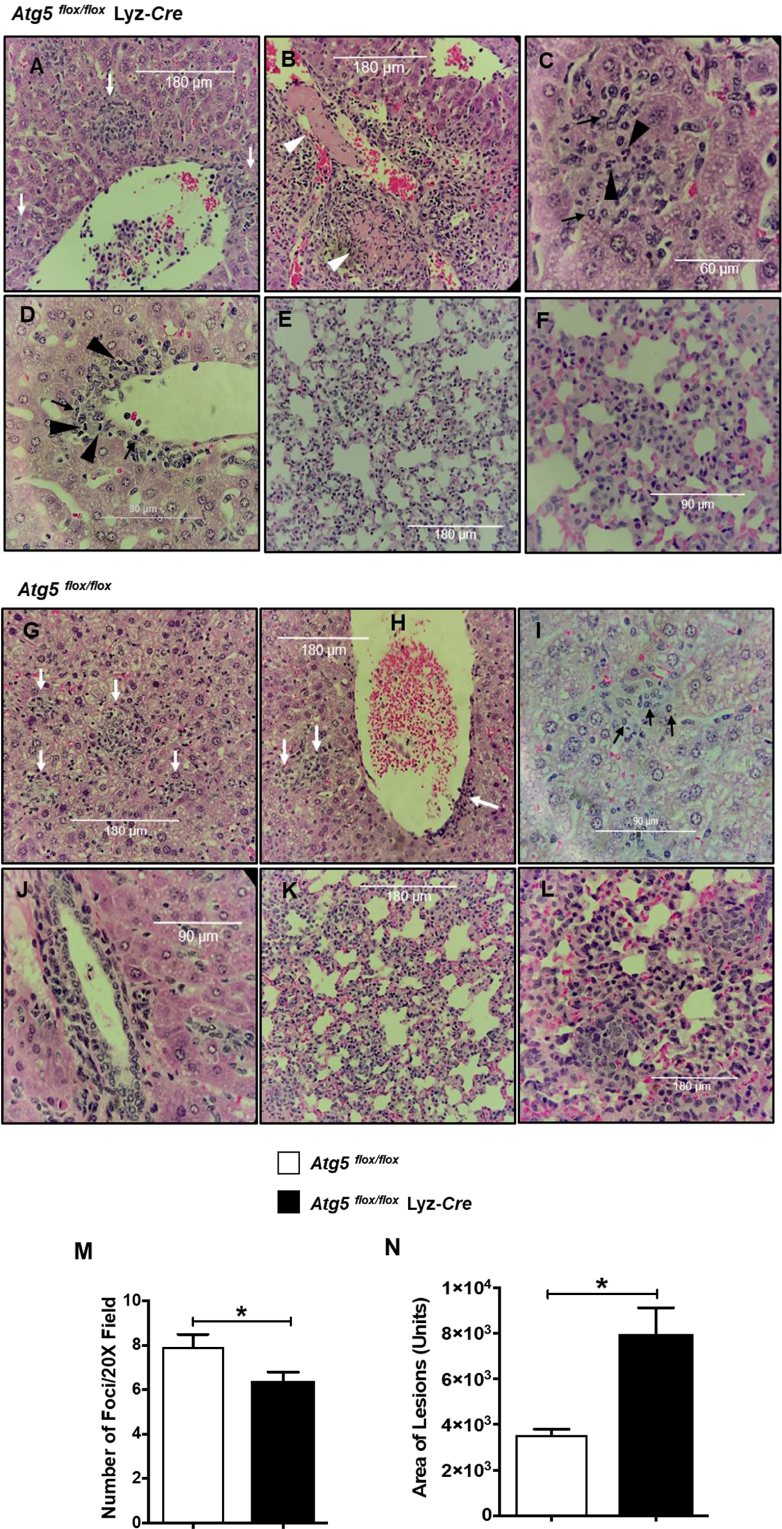
(previous Figure 7). Inflammatory cellular accumulation upon infection with *R. australis* in tissues of *Atg5 ^flox/flox^* mice and *Atg5 ^flox/flox^* Lyz-*Cre* mice. Mice were infected i.v. with *R. australis* (3 × 10^5^ PFU per mouse). On day 4 p.i., mice were sacrificed, and tissues were isolated and analyzed. Histological analysis of livers and lungs from infected *Atg5 ^flox/flox^* Lyz-*Cre* mice **(A–F)** and *Atg5 ^flox/flox^* mice **(G–L)**. Foci of inflammatory infiltration are indicated by white arrows. Thrombus or necrotic cells related to thrombosis is shown as white arrowheads **(B)**. Polymorphonuclear neutrophils (PMNs) (black arrowheads) and macrophages (black arrows) are shown in livers. Furthermore, the size **(M)** and frequency **(N)** of inflammatory lesions in livers were analyzed using ImageJ (magnification, ×20). Images were taken using an Olympus BX41 photomicroscope (Olympus America, Inc., Center Valley, PA) or using a Revolution Microscope and an iPad Pro^®^ tablet (Echo Laboratory, San Diego, CA). *p<0.05.

Histologic analysis of the H&E-stained liver and lung sections of infected animals showed inflammatory cell infiltrations and lesions in both *R. australis*-infected *Atg5 ^flox/flox^* and infected *Atg5 ^flox/flox^* Lyz-*Cre* mice on day 4 p.i. compared to uninfected controls (**Figure 2**). Livers of infected *Atg5 ^flox/flox^* Lyz-*Cre* mice showed numerous perivascular foci of inflammatory infiltration either around the central vein and portal triad or in the lobules (**Figure 2A**). Compared to *Atg5 ^flox/flox^* mice, deficiency in *Atg5* in macrophages resulted in thrombosis and infarction in the liver (**Figure 2B**). In the liver of infected *Atg5 ^flox/flox^* Lyz-*Cre* mice, inflammatory foci were randomly distributed throughout the tissue. These cellular infiltrations consisted of mainly macrophages, but also lymphocytes and neutrophils (**Figures 2C, D**). Furthermore, *R. australis*-infected *Atg5 ^flox/flox^* Lyz-*Cre* mice showed interstitial pneumonia in the lungs (**Figures 2E, F**).

Evaluation of histopathological changes in livers of infected *Atg5^flox/flox^* mice showed cellular infiltration in widely distributed foci consisting of macrophages, but fewer or no neutrophils compared to *Atg5 ^flox/flox^* Lyz-*Cre* mice (**Figures 2G–J**). The lungs of *R. australis*-infected *Atg5 ^flox/flox^* mice also showed interstitial pneumonia (**Figures 2K, L**). Interestingly, the frequency of pathological lesions was significantly reduced in livers of infected *Atg5 ^flox/flox^* Lyz-*Cre* mice compared to *Atg5 ^flox/flox^* mice (**Figure 2M**). In contrast, the inflammatory lesions in the livers of infected *Atg5 ^flox/flox^* Lyz-*Cre* mice were significantly greater in size compared to infected *Atg5 ^flox/flox^* mice (**Figure 2N**). Considering the greater *R. australis* load in tissues of *Atg5 ^flox/flox^* mice vs *Atg5 ^flox/flox^* Lyz-*Cre* mice, these results suggest that *R. australis* subverts host innate immunity *via Atg5* to support their infection in association with histopathological changes featured by increased frequency and reduced size of cellular infiltrations consisting of few or no neutrophils.

### *Atg5*-Dependent Gene Regulation by *R. australis* Infection in Macrophages

**Figure 3:**
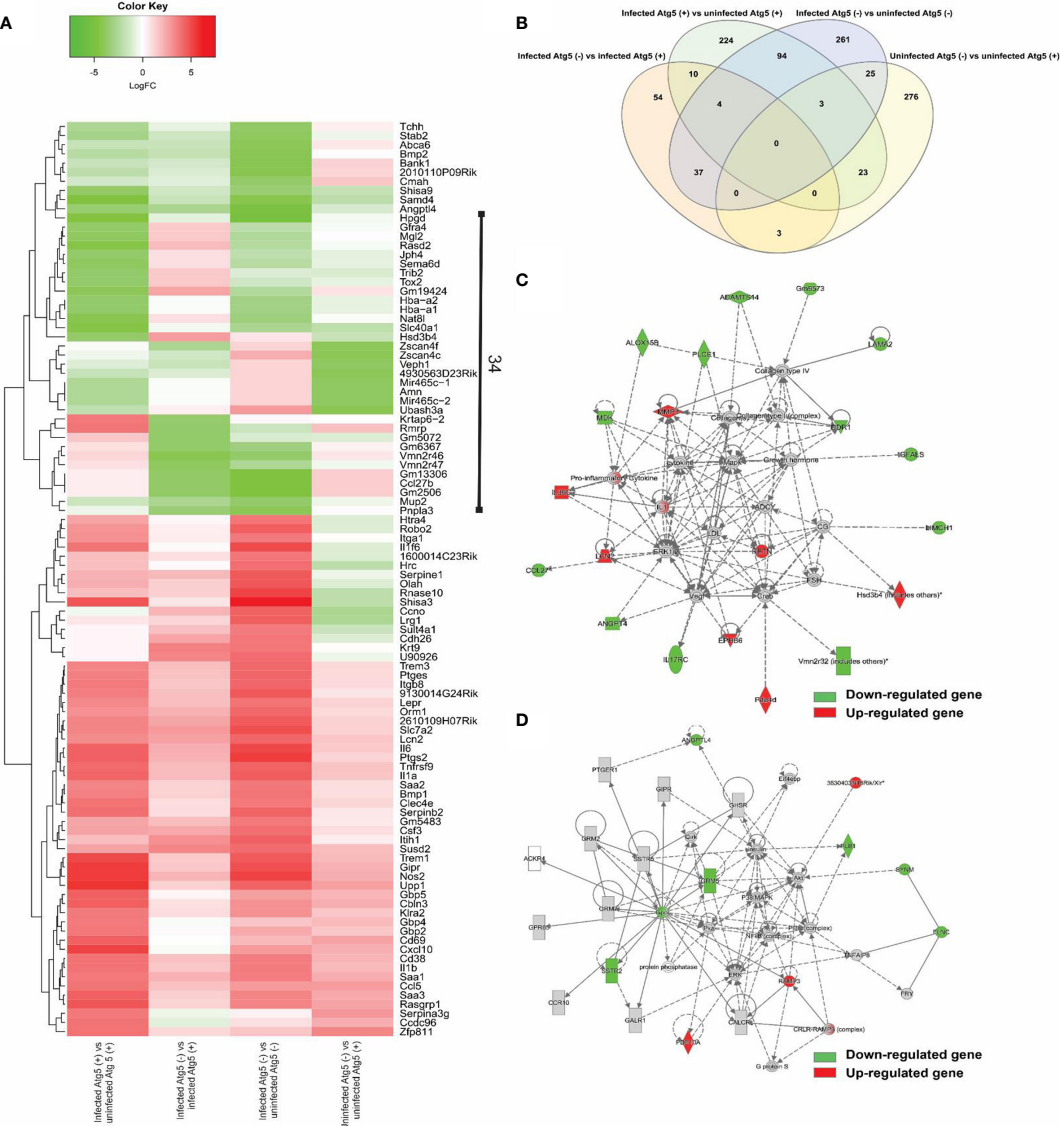
(previous Figure 3). Comparative transcriptional analysis of *R. australis*-infected *Atg5 ^flox/flox^* Lyz-*Cre* and *Atg5 ^flox/flox^* BMMs. BMMs were isolated from *Atg5 ^flox/flox^* Lyz-*Cre* and *Atg5 ^flox/flox^ mice*, and then infected with *R. australis* at an MOI of 5. At 24 h p.i., cells were collected and total RNA was extracted. RNA-seq analysis was performed as described in *Materials and Methods*. *Atg5* (+), *Atg5 ^flox/flox^* ^;^ *Atg5* (-), *Atg5 ^flox/flox^* Lyz-*Cre.* **(A)**, Heatmap and hierarchical clustering of the top 100 genes differentially regulated by *Atg5* during *R. australis* infection in mouse macrophages. The expression levels of genes are indicated by the color bar above the heatmap. Red color indicates the increased expression whereas green color indicates the decreased expression in four comparisons. **(B)**, Venn diagram showing overlap of significantly modulated genes for each of the four comparisons. **(C, D)**, IPA molecular networks analysis of differentially expressed genes in IL-1 family cytokines signaling and PI3K-Akt-mTOR signaling in *R. australis*-infected *Atg5 ^flox/flox^* Lyz-*Cre* BMMs vs *R. australis*-infected *Atg5 ^flox/flox^* BMMs. Red, up-regulated; green, down-regulated.

No prior studies have examined the *Atg5*-dependent regulation of host gene expression by *Rickettsia* in macrophages. We performed four comparisons in this study, including infected *Atg5* (+) vs uninfected *Atg5* (+), infected *Atg5* (-) vs infected *Atg5* (+), infected *Atg5* (-) vs infected *Atg5* (+), and uninfected *Atg5* (-) vs uninfected *Atg5* (+). As shown in **Figure 3**, *Atg5* (-) refers to *Atg5 ^flox/flox^* Lyz-*Cre* while *Atg5* (+) refers to *Atg5 ^flox/flox^*. First, a list of the top 100 differentially expressed genes in each comparison was used to generate a heat map (**Figure 3A**). Approximately half of these 100 genes were upregulated in infected macrophages in all four comparisons, notably pro-inflammatory cytokines (IL-1 family of cytokines and TNF-alpha) and chemokines (CCL5 and CXCL10), that may be of particular interest for the purpose of our studies. Moreover, 34 genes were differentially expressed in each comparison, suggesting that the expression levels of these genes are closely associated with *Atg5*-dependent autophagy and/or *R. australis* infection (**Figure 3A**). We generated a Venn diagram showing the overlap of significantly modulated genes among four comparisons InteractiVenn (**Figure 3B**). No gene was shared among the four comparisons. The majority of modulated genes were unique to either infected *Atg5* (-) vs uninfected *Atg5* (-), infected *Atg5* (+) vs uninfected *Atg5* (+), or uninfected *Atg5* (-) vs uninfected *Atg5* (+).

The focus of the present study is on the subversion of the host response in macrophages by *R. australis* observed in an *Atg5*-competent vs. *Atg5*-compromised mice. The networks generated by IPA analysis illustrate the interrelationships between genes and the temporal changes in gene modulation. Two molecular networks, in which IL-1 family cytokines (**Figure 3C**) and PI3K-Akt-mTOR (**Figure 3D**) were central hub molecules, were identified by IPA. The first network (**Figure 3C**) consists of 22 genes differentially expressed in infected *Atg*5 (-) and infected *Atg5* (+) BMMs. In IL-1 family cytokine signaling, nine and thirteen genes were up- and down-regulated, respectively, by *R. australis* infection in *Atg5*-deficient macrophages compared to *Atg5*-competent macrophages. Thus, *Atg5* specifically down-regulated nine genes during *R. australis* infection, including IL-1, IL-36G, lipocalin-2 (LCN2), resistin (RETN), NADPH-dependent 3-keto-steroid reductase (Hsd3b4), matrix metallopeptidase 3 (MMP3), and ephrin type-B receptor 6 (EPHB6), some of which have been shown to contribute to host innate immunity against infections (40). Therefore, *Atg5* benefits *R. australis* infection in macrophages, at least in part in association with inhibiting the members of IL-1 family cytokines in the host innate immune system. In **Figure 3D**, the hub molecules are PI3K, Akt, insulin, and eukaryotic initiation factor 4E-binding protein 1 (Eif4ebp) (41). When mTORC1 is active, it phosphorylates (activates) p70S6 kinase (S6K) and the eIF4e binding protein (42). Thus, the PI3K-Akt-mTOR signaling pathway was regulated by *R. australis* infection. Compared to infected *Atg5 ^flox/flox^* BMMs, 4 genes including RAMP3 and PDE10A were upregulated while 7 genes including ANGPTL4 and Gpcr were downregulated in infected *Atg5 ^flox/flox^* Lyz-*Cre* BMMs by *R. australis* infection (**Figure 3D**). These results suggest that the enhanced *R. australis* infection in *Atg5*-competent macrophages is associated with the alterations of the key cellular signaling pathway, PI3K-Akt- mTOR.

### *R. australis* Suppresses the Production of Inflammatory Cytokines by Infected Macrophages *via* a Modified *Atg5*-Dependent Autophagic Response

**Figure 4:**
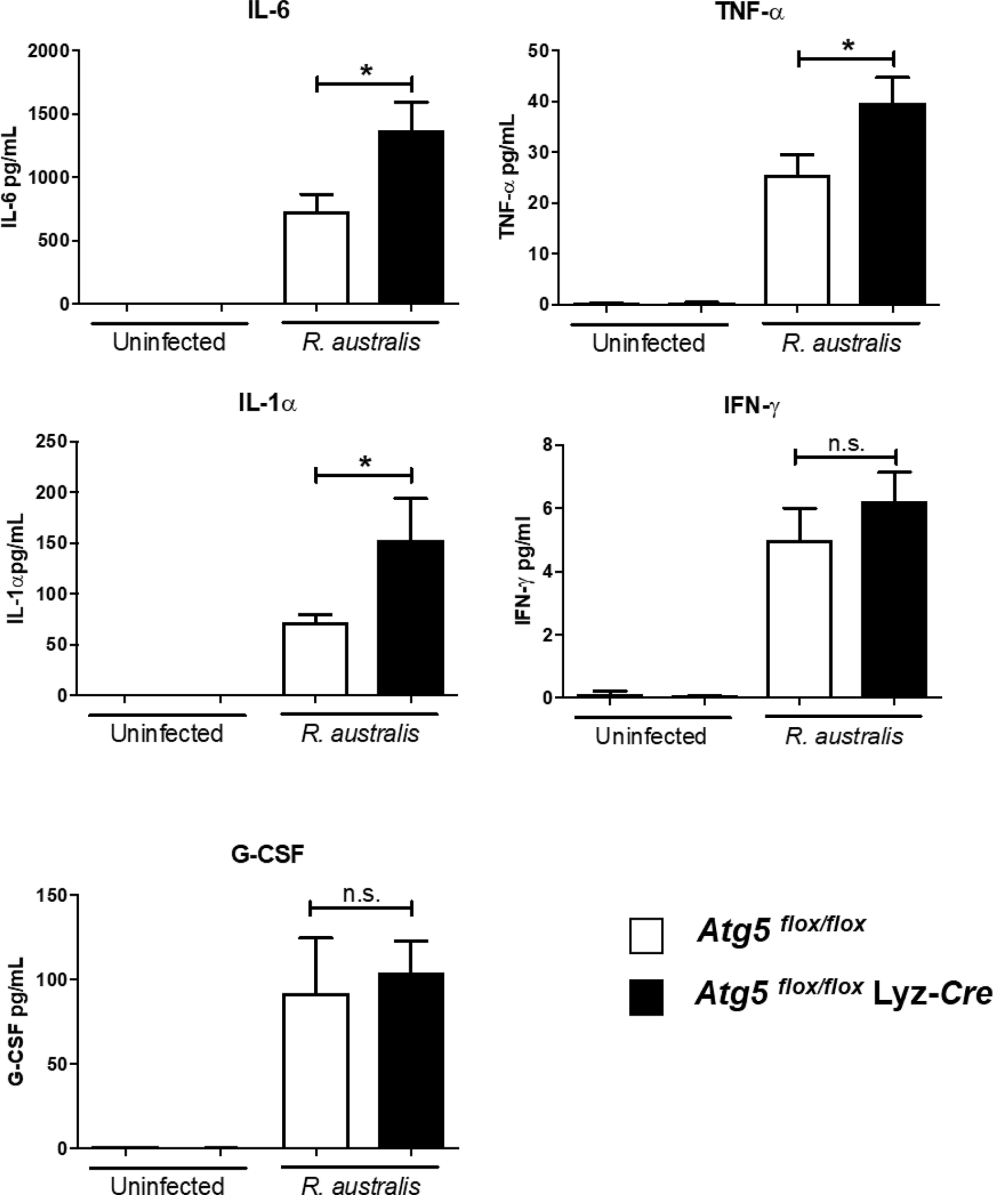
(previous Figure 4). *R. australis* counteracts the production of pro-inflammatory cytokines by infected macrophages *via Atg5*-dependent autophagy. BMMs were isolated from *Atg5 ^flox/flox^* Lyz-*Cre* and *Atg5 ^flox/flox^* mice, and they were infected with *R. australis* at an MOI of 5. At 24 h p.i., supernatant was harvested. Production levels of cytokines including IL-6, TNF-α, IL-1α, IFN-γ and G-CSF in the supernatant were assessed by Bioplex assay. Results are means ± SE of data from three independent experiments. *p<0.05; n.s., not statistically significant.

Our recently published studies have shown that *R. australis* induces a modified *Atg5*-dependent autophagic response to benefit its replication through inhibiting the secretion of rickettsicidal IL-1β, although IL-1β-independent mechanisms also contribute to this process (4). To explore IL-1β-independent factors involved in supporting *R. australis* infection in macrophages, we measured the secretion levels of five cytokines in *R. australis*-infected *Atg5 ^flox/flox^* Lyz-*Cre* BMMs and *Atg5 ^flox/flox^* BMMs at 24 h p.i. Uninfected BMMs of *Atg5*-conditional knockout mice did not produce any significant levels of the examined cytokines (**Figure 4**). The deficiency of *Atg5*-dependent autophagy did not change the levels of either IFN-γ or G-CSF secreted by infected macrophages (**Figure 4**). Interestingly, *R. australis*-infected *Atg5 ^flox/flox^* Lyz-*Cre* BMMs produced significantly greater levels of IL-6, IL-1α, and TNF-α compared to infected *Atg5 ^flox/flox^* BMMs (**Figure 4**). The enhanced production of IL-6, IL-1α and TNF-α was associated with reduced *R. australis* infection in *Atg5*-deficient macrophages (4). These results suggest that *R. australis* exploits the *Atg5*-dependent autophagic response to inhibit the production of IL-6, IL-1α, and TNF-α in macrophages to support their infection.

**Figure 5:**
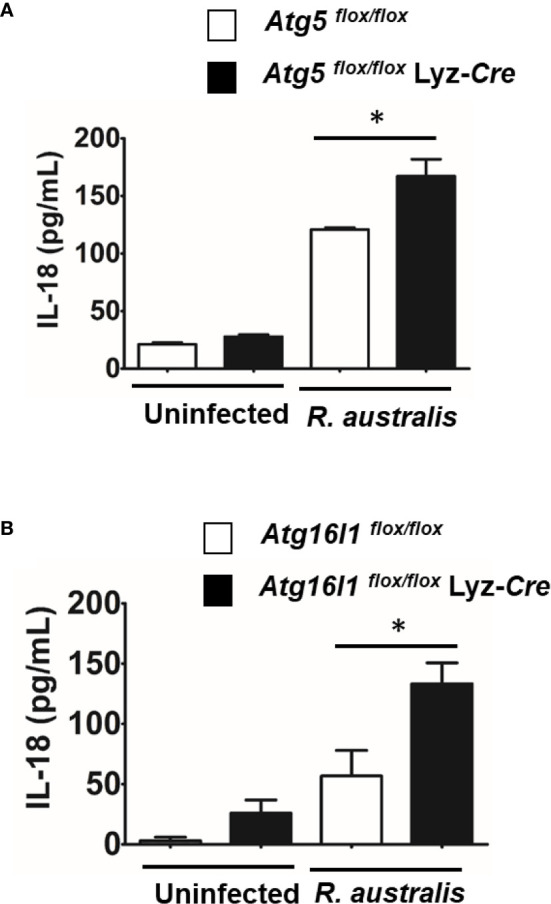
(previous Figure 5). *R. australis* suppressed the production of IL-18 by infected macrophages *via Atg5*-dependent autophagy. BMMs were isolated from *Atg5 ^flox/flox^* Lyz-*Cre* and *Atg5 ^flox/flox^* mice, and *Atg16l1 ^flox/flox^* Lyz-*Cre* and *Atg16l1 ^flox/flox^* mice. Macrophages were infected with *R. australis* at an MOI of 5. At 24 h p.i., supernatant was harvested. Concentrations of IL-18 in the supernatant of infected *Atg5 ^flox/flox^* Lyz-*Cre* BMMs and *Atg5 ^flox/flox^* BMMs **(A)**, and infected *Atg16l1 ^flox/flox^* Lyz-*Cre* BMMs and *Atg16l1 ^flox/flox^* BMMs **(B)** were assessed by ELISA. Results are means ± SE of data from two independent experiments. *p < 0.05.

IL-18 is an important member of the IL-1 family of cytokines (43–45). To determine the effect of autophagy on production of IL-18 by *R. australis*-infected macrophages, we employed BMMs from both *Atg5-* and *Atg16l1-*conditional knockout mice. *R. australis* infection resulted in significantly greater production levels of IL-18 in *Atg5 ^flox/flox^* Lyz-*Cre* BMMs compared to *Atg5 ^flox/flox^* BMMs (**Figure 5A**). Furthermore, the secretion levels of IL-18 by *R. australis*-infected *Atg16l1 ^flox/flox^* BMMs were significantly less than those by infected *Atg16l1 ^flox/flox^* Lyz-*Cre* BMMs (**Figure 5B**). We had demonstrated that deletion of *Atg16l1* significantly reduces the concentrations of *R. australis* (4). Thus, the reduced IL-18 production by *Atg16l1 ^flox/flox^* BMMs was associated with a greater rickettsial load in these BMMs compared to *Atg16l1 ^flox/flox^* Lyz-*Cre* BMMs. These results clearly revealed that *R. australis* suppressed the production of IL-18 in an autophagy-dependent manner, consistent with the results of *Atg5*-dependent regulation of IL-1 family cytokines at the transcriptional level by this bacterium, as shown in **Figure 3C**.

### *R. australis* Induces Activation of mTORC1 Signaling

**Figure 6:**
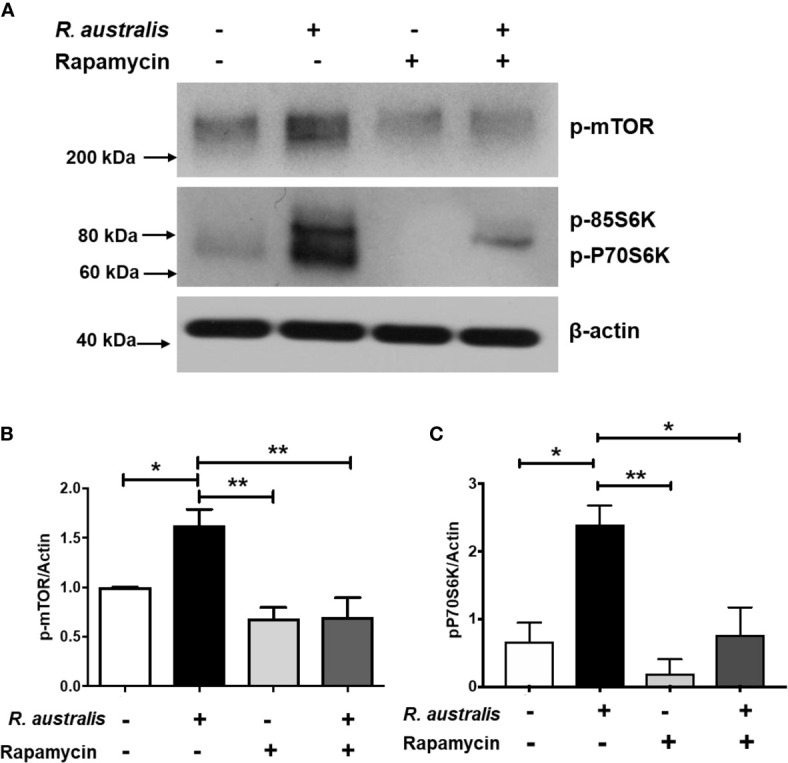
(previous Figure 1). *R. australis* activates mTORC1 in mouse macrophages. BMMs of WT B6 mice were isolated and infected with *R. australis* at an MOI of 5. To inhibit mTORC1 signaling, cells were treated with 50 ng/mL of rapamycin for 4 hours prior to infection with *R. australis*. At 1 h p.i., cells were collected, and cell lysates were immunoblotted with antibodies directed against phosphorylated mTOR, phosphorylated p70S6K, and β-actin **(A)**. The activation of phosphorylated mTOR **(B)** and phosphorylated p70S6K **(C)** was analyzed by densitometry using β-actin as a normalization control with three independent replicates. *p < 0.05; **p < 0.01.

As described above (**Figure 3D**), *R. australis* differentially regulated the genes in the PI3K-Akt-mTOR signaling pathway in macrophages *via* an *Atg5*-dependent mechanism. Since inhibition of mTORC1 by rapamycin treatment promotes *R. australis* infection (4), mTORC1 activation is likely important to regulate the cellular environment during rickettsial infection. This was the impetus to study whether *R. australis* infection has an impact on mTORC1 signaling and if mTORC1 signaling regulates autophagy induction. To address these questions, we infected B6 BMMs with *R. australis* and determined the phosphorylation of mTOR and P70S6 kinase as a read-out for mTORC1 activity. Surprisingly, *R. australis* infection induced direct phosphorylation of mTOR on serine 2448 and phosphorylation of P70S6 kinase (threonine 389) in B6 BMMs compared to uninfected controls at as early as 1 h p.i. when autophagic response is induced by these bacteria (**Figure 6A**) (4). Densitometry analysis from three independent experiments showed that the expression levels of phosphorylated mTOR and P70S6 in *R. australis*-infected macrophages were significantly greater than in uninfected macrophages (**Figures 6B, C**). These results suggest that infection with *R. australis* leads to both direct and indirect activation of mTORC1 in infected macrophages (4). To confirm that the increase in phosphorylated mTOR and P70S6 kinase represents the activation of mTORC1 signaling, B6 BMM macrophages were pre-treated with rapamycin prior to infection. Blockage of mTORC1 signaling by treatment with rapamycin for less than 6 h nearly abolished the increase in both phosphorylated mTOR and P70S6 (**Figure 6**). Thus, these results demonstrated that *R. australis* infection activated mTORC1 signaling in mouse macrophages, which can be effectively eliminated by treatment with rapamycin.

### *R. australis* Induces Autophagic Response While Activating mTORC1 Signaling

**Figure 7:**
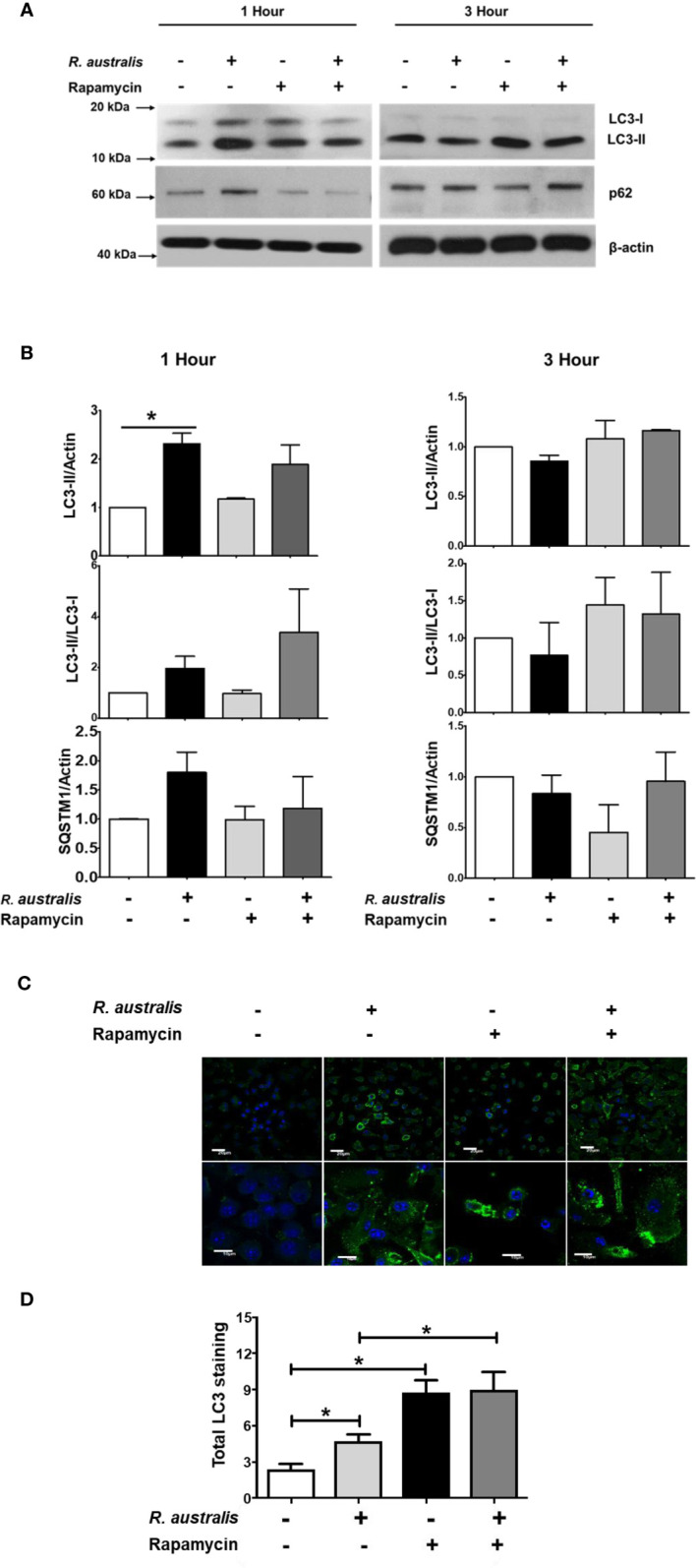
(previous Figure 2). Interactions of mTORC1 and autophagy with *R. australis* in macrophages. BMMs of WT B6 mice were isolated and infected with *R. australis* at an MOI of 5. To inhibit mTORC1 signaling, cells were treated with 50 ng/mL of rapamycin for 4 hours prior to infection with *R. australis*. Cells were collected at 1 h and 3 h p.i., and cell lysates were immunoblotted with antibodies directed against LC3-II, p62 and β-actin **(A)**. **(B)**, The ratios of LC3-II/Actin, LC3-II/LC3-I, and SQSTM1/Actin in uninfected and infected BMMs with or without rapamycin treatment at 1 h and 3 h p.i. were analyzed by densitometry. **(C)**, Representative confocal immunofluorescence microscopic images of uninfected and infected BMMs with or without rapamycin treatment at 1 h p.i. at a magnification of 20x. Green, LC3 puncta; blue, nuclei (DAPI). Bar = 20 µm in the upper and 10 µm in the bottom row, respectively. **(D)**, Total LC3 staining was quantified using Image J software. Microscopy data represent two to three independent experiments. Data shown are mean ± SE. Group comparison was not labeled if not statistically significant. *p < 0.05.

Our previous studies have reported that *R. australis* induces ATG5 (+) LC3 (+) autophagosomes with less likely degradative autophagy in macrophages at 1 h p.i (4). As shown in **Figure 6**, *R. australis* activated mTORC1 at the same time when *Atg5*-dependent autophagic response was induced in the same type of cells. This is interesting to us because mTOR-dependent autophagy would have decreased phosphorylation of mTOR. To this end, we stimulated B6 BMMs with rapamycin in the context of *R. australis* infection and analyzed autophagy induction by examining the levels of LC3-II, SQSTM1 and the conversion of soluble LC3-I to lipid bound LC3-II. At 1 h p.i., consistent with our previous report, *R. australis* alone induced significantly increased levels of LC3-II compared to uninfected controls without a significantly reduced level of SQSTM1/p62, indicating a modified autophagic response (**Figures 7A, B**). In contrast, rapamycin treatment slightly increased levels of LC3-II/LC3-I and slightly reduced levels of SQSTM1/p62 in *R. australis*-infected macrophages at 1 h p.i. (**Figures 7A, B**). However, these changes were not statistically significant (**Figure 7B**). At 3 h p.i., we did not find any significant change in expression levels of LC3-II, LC3-II/LC3-I conversion, or SQSTM1/p62 in *R. australis*-infected macrophages treated with rapamycin compared to controls (**Figures 7A, B**). Since *R. australis* is known to modify autophagy into an *Atg5*-dependent autophagic response (4), we decided to determine the quantity of LC3 (+) autophagosomes by immunofluorescence confocal microscopy. Indeed, at 1 h p.i., LC3 puncta staining was significantly increased in *R. australis*-infected BMMs treated with rapamycin compared to macrophages infected with rickettsiae alone (**Figures 7C, D**). However, *R. australis*-infected macrophages with rapamycin treatment did not show significantly increased LC3-II fluorescence intensities compared to rapamycin-treated uninfected cells. In other words, we did not observe synergistic or additive effects by *R. australis* infection in rapamycin-treated macrophages at 1 h p.i. (**Figures 7C, D**). Thus, inhibition of mTORC1 most likely enhanced the accumulation of autophagosomes in *R. australis*-infected macrophages. These data suggest that *R. australis* induces a modified mTORC1-independent autophagic response in macrophages.

## Discussion

**Figure 8:**
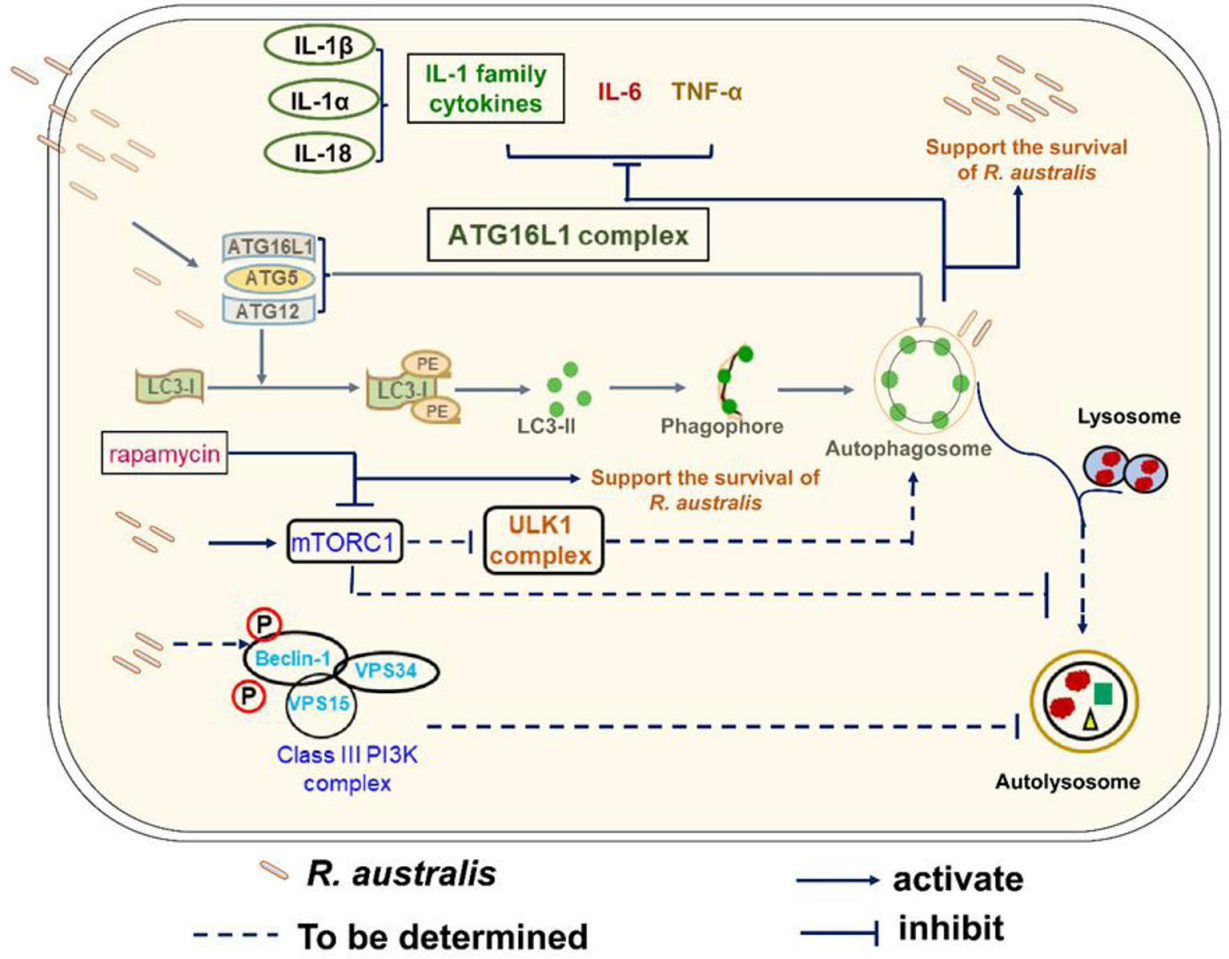
Schematic diagram of interactions of *Atg5*-dependent autophagy and mTORC1 with *R. australis* in macrophages. This schematic diagram depicts the molecular mechanisms involved in the modulation of autophagy and activation of mTORC1 by *R. australis* in order to benefit bacterial infection. *R. australis* activates the ATG5-ATG12-ATG16L1 complex leading to accumulation of LC3 (+) autophagosomes. *Atg5* (+) LC3 (+) autophagosomes induced by *R. australis* in macrophages inhibit production levels of IL-1 family cytokines including IL-1β, IL-1α and IL-18 as well as other inflammatory cytokines such as IL-6 and TNF-α, the effects of which favor rickettsial infection. *R. australis* activates mTORC1 signaling at 1 h p.i. in macrophages when the *Atg5*-dependent autophagic response is induced. Thus, *R. australis*-induced autophagic response is resistant to regulation by mTORC1 signaling. It would be interesting to explore whether *R. australis*-induced autophagosomes can fuse with lysosome and mature into autolysosomes, and how ULK and class III PI3K complex are involved in this process in the future. Solid lines represent pathways demonstrated in our current and previous studies, while dashed lines refer to the mechanisms hypothesized but not tested.

Our recent report on interactions of autophagy with *R. australis* paved the way for the present study (4). Autophagy is a fundamental cellular homeostasis program that degrades surplus cellular contents in cytoplasm to provide sources of energy (46). Increasing evidence has demonstrated various mechanisms by which host autophagy interacts with invading microbial pathogens. However, the interplay of autophagy with the host inflammatory response to infectious agents *in vivo* and the subsequent pathological changes in tissues are not well understood. Studies revealing such roles of autophagy are likely to provide insightful information toward understanding the pathogenesis of these infectious diseases and the potential development of novel therapeutics targeting autophagy. In this regard, our studies demonstrated several novel findings. *R. australis*, a member in the transitional group of the *Rickettsia* genus, was revealed to activate mTORC1 signaling in primary mouse macrophages. Moreover, mTORC1 control of autophagy was dysregulated during *R. australis* infection at least at 1 h p.i. (**Figure 8**). Our studies also demonstrated that deficiency of *Atg5* enhanced the host control of *R. australis in vivo* accompanied by numerous infiltrations of inflammatory cells in tissues, including neutrophils. *R. australis* infection significantly regulated mammalian host genes and subverted host innate inflammatory responses to support the infection by modifying *Atg5*-dependent autophagy.

The mTOR signaling pathway is widely expressed in tissues and cells of mammalian hosts, and functions as an evolutionarily conserved sensor of environmental and endogenous stress (47). The mTOR is a down-stream effector of PI3K-Akt signaling pathway and mediates nutrient-dependent intracellular signaling related to cell growth, proliferation, and differentiation (16, 48). Hepatitis C virus (HCV) activates mTOR *via* the viral nonstructural protein 5A (NS5A) to enhance cell survival by blocking apoptosis (49). Human cytomegalovirus (HCMV) induces mTOR activation and maintains this activation throughout infection (50). Zullo and Lee reported that several *Mycobacterium* species activate mTOR signaling and induce autophagy; however, they show that the induction of autophagy in macrophages is mTOR-independent (51). Rapamycin has been reported to mainly inhibit mTORC1, particularly within 24 hour of stimulation (52, 53). In the present study, treatment with rapamycin for 5 hours nearly abolished the increase in phosphorylated mTOR and P70S6 in *R. australis*-infected macrophages (**Figures 6** and **7**), suggesting activation of mTORC1 signaling by rickettsiae. Although the results in **Figures 6** and **7** provided both direct and indirect evidence that *R. australis* activates mTORC1 signaling, this conclusion can even be further supported by the unaltered total expression levels of mTOR and P70S6 in these infected macrophages. We are currently performing such experiments in our laboratory. Furthermore, it remains unknown whether *R. australis* activates mTORC2 signaling or not. It is not surprising to us that *R. australis* dysregulated the control of autophagy by mTORC1 in macrophages at 1 h p.i. based on two findings previously reported (4, 54). First, *R. australis* does not induce a canonical autophagy pathway characterized by increased LC3-II and reduced p62 (4). Instead, *R. australis* manipulates an *Atg5*-dependent autophagic response in order to facilitate their infection (**Figure 8**). Secondly, given the recent discovery that *R. typhi* activates both Class I and III PI3Ks (54), it is interesting to speculate that *R. australis* activates class III PI3K complex to further trigger the activation of mTORC1 (**Figure 8**). Increasing evidence indicates that mTORC1 also directly regulates the subsequent steps of the autophagy process, including the nucleation, autophagosome elongation, autophagosome maturation and termination (16). It is, therefore, worthwhile to investigate whether and how mTORC1 is involved in regulating the accumulation of autophagosomes and maturation of autophagosome into autolysosome during *R. australis* infection. Furthermore, previous studies have demonstrated that inhibition of apoptosis is essential for endothelial cell survival during *R. rickettsii* infection. *R. rickettsii* even protects host endothelial cells from staurosporine-induced cell death (55, 56). We also reported that *R. australis* does not induce cell death in infected mouse BMMs (5). Activation of mTOR is known to promote cell survival (57). Thus, it is an attractive hypothesis that the activation of mTOR, including mTORC1 and/or mTORC2 signaling, in cells infected with rickettsiae may serve as a mechanism for promoting cell survival.

Autophagy has been shown to negatively regulate host inflammatory cytokines, particularly the IL-1 family of cytokines. *R. australis* inhibits IL-1β secretion in infected BMMs *in vitro* and in sera by modulating *Atg5*-dependent autophagy (4). *In vitro* neutralization of IL-1β by specific antibodies abolishes the enhanced *R. australis* infection by an *Atg5*-dependent autophagic response in macrophages (4). *R. australis* has been reported to induce significant levels of IL-18, another member in IL-1 family cytokines, by B6 BMMs (5). The differences in production levels of IL-18 by BMMs of *Atg5*- and *Atg16l1*-conditional knockout mice (**Figure 5**) from those by B6 BMMs could result from the variabilities in mouse genetic background. Interestingly, IL-1, IL-2, IL-6, TNF-α, IFN-γ, and TGF-β are known to induce autophagy while IL-4, IL-10 and IL-13 inhibit autophagy (58). Our present study demonstrated autophagy-dependent regulation of IL-1α, IL-6, IL-18, and TNF-α in murine macrophages infected with *R. australis in vitro* (**Figures 4** and **5**). These inflammatory cytokines upregulated by *Atg5*-dependent autophagy may contribute to reduced rickettsial loads in tissues of *R. australis*-infected *Atg5*-deficient macrophages (4). However, *R. australis* primarily targets microvascular endothelial cells *in vivo* while macrophages are the initial target cells at the cutaneous entry site of rickettsiae (3, 59, 60). It is not surprising to see a difference in cytokine profiles in *R. australis*-infected BMMs and sera of *R. australis*-infected *Atg5-*conditional knockout mice. For example, macrophages are not a major producer of IFN- *γ*, and our *in vitro* data confirm this conclusion (**Figure 4**). In contrast, in *Rickettsia*-infected mice, NK and T cells are both the major cells secreting IFN-*γ* (28, 31, 38, 39). Although an effective Th1-type cytokine response is critical for controlling rickettsial infection as demonstrated by us and other groups (28, 31, 38, 39, 61–63), the serum of *R. australis*-infected *Atg5 ^flox/flox^* Lyz-*Cre* mice had significantly lower levels of IFN-γ, with lower bacterial loads in tissues, compared to *Atg5 ^flox/flox^* mice (**Figure 6**) (4). The lower level of IFN-*γ* in the serum of *Atg5 ^flox/flox^* Lyz-*Cre* mice compared to *Atg5 ^flox/flox^* animals may result from reduced rickettsial loads in the tissues. Another explanation is that the enhanced systemic production of IFN-γ in *Atg5 ^flox/flox^* mice may result from robust activation of NK and T cells by autophagy-competent macrophages in host innate immunity. Overall, *R. australis* ultimately subverted the host innate inflammatory response, which favored its own infection *in vivo*.

The histopathological data of *R. australis*-infected *Atg5*-conditional knockout mice showed that *Atg5 ^flox/flox^* Lyz-*Cre* mice had significantly fewer pathological foci with larger size in liver compared to *Atg5 ^flox/flox^* mice (**Figure 2**). We hypothesize that the pathological changes in infected *Atg5 ^flox/flox^* Lyz-*Cre* mice represent effective host control of pathogenic *Rickettsia* in infected tissues resulting from recruiting a number of potent innate immune cells. In contrast, the pathological changes in *R. australis*-infected *Atg5 ^flox/flox^* mice represent paralysis or subversion of innate host immunity, as manifested by the greater frequency of foci but a limited area of cellular infiltration. Our *in vivo* pathology results strongly suggest that *Atg5*-deficiency promotes an inflammatory response consisting of a considerable frequency of neutrophils. IL-1β is a major stimulator of leukocyte recruitment through its ability to up-regulate adhesion to endothelial cells (64, 65). *R. australis* infection induces significantly greater levels of IL-1β in the serum of infected *Atg5 ^flox/flox^* Lyz-*Cre* mice vs *Atg5 ^flox/flox^* mice (4). The accumulation of neutrophils in the inflammatory foci in liver of infected *Atg5 ^flox/flox^* Lyz-*Cre* mice was possibly associated with the greater production levels of IL-1β compared to infected *Atg5 ^flox/flox^* mice.

This study revealed that *R. australis* induced *Atg5*-dependent autophagic response while activating mTORC1 signaling in macrophages. Additionally, our data demonstrated that *R. australis* modulated *Atg5*-dependent autophagy to inhibit inflammatory cytokines at both transcriptional and post-transcriptional levels in macrophages, including IL-6, IL-1α, TNF-α, and IL-18. In conclusion, the *R. australis*-modified autophagic response in macrophages supports the *in vivo* infection through subversion of host innate immunity against rickettsiae.

## Data Availability Statement

The data has been uploaded to the GEO, with accession number GSE171160.

## Ethics Statement

The animal study was reviewed and approved by the Institutional Animal Care and Use Committee at UTMB.

## Author Contributions

JB and CR performed the experiments. JB and RF wrote the manuscript. JB, CR, and RF designed the experiments and collected data. DW performed the histopathological analysis as a board-certified pathologist, analyzed the data, and revised the manuscript. SW performed the RNA-Seq and data analysis. KK analyzed the RNA-Seq results by IPA analysis and generated **Figure 3**. All authors contributed to the article and approved the submitted version.

## Funding

This work was supported by grant AI133359 to RF from the National Institute of Allergy and Infectious Diseases. JB was supported by the National Institutes of Health T32 Training Grant (AI060549) at the University of Texas Medical Branch.

## Conflict of Interest

The authors declare that the research was conducted in the absence of any commercial or financial relationships that could be construed as a potential conflict of interest.
